# Prognostic importance of direct assignment of parent of origin via long-read genome and epigenome sequencing in retinoblastoma

**DOI:** 10.1172/jci.insight.188216

**Published:** 2024-12-26

**Authors:** Andrew W. Stacey, Kenji Nakamichi, Jennifer Huey, Jeffrey Stevens, Natalie Waligorski, Erin E. Crotty, Russell N. Van Gelder, Debarshi Mustafi

**Affiliations:** 1 Department of Ophthalmology and Roger and Angie Karalis Johnson Retina Center, University of Washington, Seattle, Washington, USA.; 2 Division of Ophthalmology, Seattle Children’s Hospital, Seattle, Washington, USA.; 3 Fred Hutch Cancer Consortium, Seattle, Washington, USA.; 4 Division of Oncology and; 5 Division of Genetic Medicine, Seattle Children’s Hospital, Seattle, Washington, USA.; 6 Division of Hematology, Oncology, Bone Marrow Transplant, and Cellular Therapy, Department of Pediatrics, Seattle Children’s Hospital, University of Washington, Seattle, Washington, USA.; 7 Ben Towne Center for Childhood Cancer Research, Seattle Children’s Research Institute, Seattle, Washington, USA.; 8 Departments of Laboratory Medicine and Pathology and Biological Structure, University of Washington, Seattle, Washington, USA.; 9 Brotman Baty Institute for Precision Medicine, Seattle, Washington, USA.

**Keywords:** Genetics, Ophthalmology, Cancer, Genetic variation, Molecular genetics

## Abstract

**BACKGROUND:**

Current clinical sequencing methods cannot effectively detect DNA methylation and allele-specific variation to provide parent-of-origin information from the proband alone. Parent-of-origin effects can lead to differential disease, and the inability to assign parent of origin in de novo cases limits prognostication in the majority of affected individuals with retinoblastoma, a hereditary cancer with suspected parent-of-origin effects.

**METHODS:**

To directly assign parent of origin in patients with retinoblastoma, we extracted genomic DNA from blood samples for sequencing using a programmable, targeted, single-molecule, long-read DNA genomic and epigenomic approach. This allowed germline variant calling and simultaneous haplotype-resolved CpG methylation in participants with familial (*n* = 7) and de novo (*n* = 9) retinoblastoma.

**RESULTS:**

Targeted long-read sequencing allowed phasing genomic variation with a differentially methylated region in intron 2 of the retinoblastoma gene to confirm parent of origin in known familial samples. This approach allowed us to directly assign parent of origin in simple and complex de novo cases from the proband alone. The ability to assign parent of origin in all retinoblastoma cases showed that harboring disease-causing variants on the paternally inherited allele, whether arising familially or de novo, was associated with more advanced cancer staging at presentation and significantly greater risk of chemotherapy failure (*P* = 0.002).

**CONCLUSION:**

This study demonstrates the diagnostic potential of multiomic long-read profiling to unveil the parent-of-origin effect in hereditary cancer. The approach in this work will be instrumental in assigning parent of origin to other genetic diseases using local and distant imprinting signals in the genome.

**FUNDING:**

National Eye Institute, NIH; Gerber Foundation; Research to Prevent Blindness; Angie Karalis Johnson Fund; Dawn’s Light Foundation; and Mark J. Daily, MD Research Fund

## Abstract 2

Long-read DNA sequencing reveals from which parent a cancer-causing variant is inherited for more precise prognosis and treatment.

## Introduction

Parent-of-origin effect can give rise to distinct clinical phenotypes depending on whether an allele is inherited maternally or paternally. The best-characterized parent-of-origin effect is genomic imprinting, an epigenetic process whereby DNA methylation preferentially silences either the maternal or paternal inherited allele (1). The most common imprinting disorders (such as Angelman, Prader-Willi, and Beckwith-Wiedemann syndromes) result from altered expression of imprinted genes (2–4). Altered expression of the imprinted genes in chromosomal region 11p15.5 causes Beckwith-Wiedemann syndrome, a syndrome that is associated with an increased risk of tumors, particularly Wilms’ tumors (5). Hypermethylation at the *IGF2*-*H19* locus in 11p15.5 (6, 7) leads to Wilms’ tumor, which is the most common solid tumor in the pediatric population. Molecular diagnosis of imprinted disorders can require multiple sequential tests in some cases (8), and short DNA reads used by current clinical sequencing modalities can limit the ability to assign parent of origin (9).

The autosomal dominant cancer syndrome, retinoblastoma (RB), is the most common pediatric intraocular cancer (10). Heritable RB is caused by a germline mutation in the retinoblastoma gene (*RB1*) (11–14) followed by a second somatic mutation (15). It has demonstrated a parent-of-origin effect with more advanced disease classification and worse clinical outcomes when hypomorphic variants in *RB1* are inherited paternally compared with maternally in known heritable cases (16–19). Parent-of-origin determination of a disease allele in affected individuals requires familial DNA samples, but in cases of de novo pathogenic variants in *RB1*, the parent of origin cannot be accurately assigned even with familial DNA samples. In heritable RB, greater than 90% of germline cases are de novo with no associated family history. Thus, prognostic information is unavailable to a majority of afflicted individuals. Furthermore, patients with germline *RB1* pathogenic variants are not only at higher risk of bilateral ocular disease but also at an increased risk of additional malignant neoplasms throughout their lives that are associated with significant morbidity and mortality (20, 21).

There is a well-established differentially methylated region (DMR) located in intron 2 of *RB1* that exhibits a parent of origin–specific methylation pattern. It is methylated on the maternal allele and unmethylated on the paternal allele (22). This could be utilized to determine parent of origin from the proband alone, including the majority of cases that arise de novo. However, current next-generation clinical sequencing methods of peripheral blood do not routinely cover the entire *RB1* locus, and even with genome sequencing of peripheral blood and tumor samples targeting *RB1* (23), identification of epigenetic changes and chromosomal phase information requires secondary analysis, limiting their ability to provide parent-of-origin diagnostics. In this work, we leverage the ability of targeted long-read sequencing to simultaneously assay genetic and epigenetic base level changes to provide haplotype-phased long-read genome and CpG methylome data in 16 cases of heritable RB in a single sequencing run. We benchmarked this multiomic approach in 7 cases with known familial inheritance of RB to show that we can determine parent of origin in each case. We then applied this approach to 9 RB cases with de novo germline variants ranging from small nucleotide variants (SNVs) to large structural variants (SVs) to resolve parent-of-origin alleles. Finally, we showed in our cohort of 16 samples that paternal origin of the germline *RB1* variant is not only associated with more severe ocular disease on clinical presentation but also prognostic for increased risk of chemotherapy failure and thus worse visual outcomes and increased risk of loss of the eye.

## Results

### Long-read sequencing allows phasing of germline variants with an imprinted signal in RB1 to confirm parent of origin in familial cases of RB.

Allele-specific methylation calling requires detection of DNA methylation and allele-specific variants on the same or linked sequencing reads. The standard for DNA methylation detection is whole-genome bisulfite sequencing (24), which was originally used to identify the imprinted region in *RB1* (22). Whereas short-read sequencing of bisulfite-treated DNA can detect methylation sites, the reads are too small to span allele-specific variants to provide chromosomal phase information. These limitations can be overcome with long-read sequencing on the Oxford Nanopore Technologies (ONT) platform, which allows real-time, single-molecule sequencing by detecting the ion current as single-stranded DNA passes through the sequencing nanopore in single-nucleotide steps (25). Moreover, the electrical current signals of 5-methylcytosine (5-mC) can be distinguished from cytosine of native DNA without specialized preparation (26, 27) to carry out simultaneous epigenomic profiling. This approach can be used to more reliably detect genome-wide, allele-specific methylation (28) and detect parent of origin without the need for parental DNA (29). Furthermore, the use of adaptive sampling can focus sequencing bandwidth to specific regions of the genome for enhanced depth of coverage for more efficient and rapid phasing of variant and methylation signals (30).

**Figure 1:**
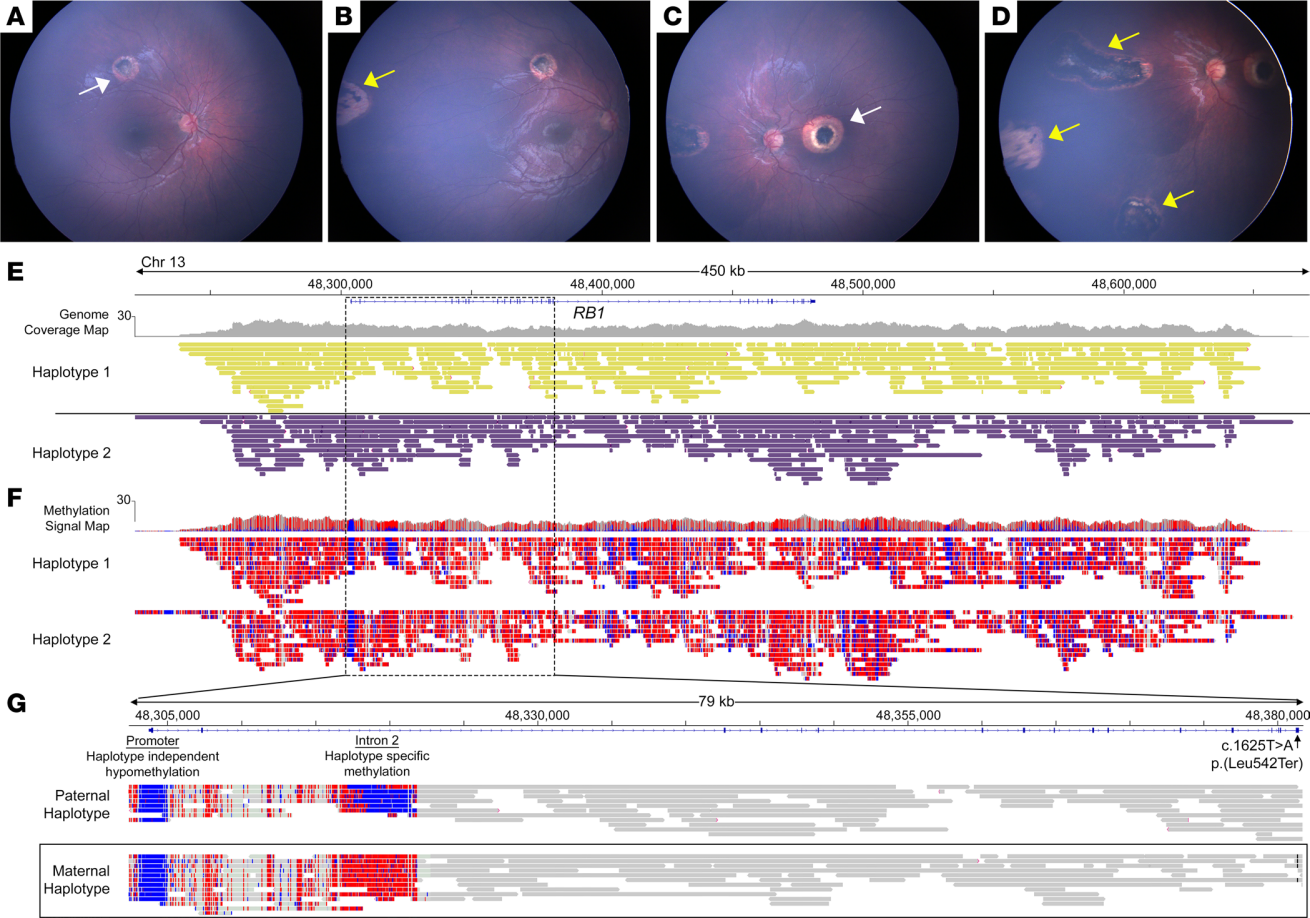
Targeted long-read sequencing allows genomic and CpG methylome profiling to identify parent of origin of a germline *RB1* variant from the proband alone. A patient with maternally inherited RB presented with macular (white arrows) and extramacular tumors (yellow arrows) in the (**A** and **B**) right and (**C** and **D**) left eyes. Targeted long-read sequencing of the *RB1* locus produced haplotyped (**E**) genomic and (**F**) epigenomic data (methylated CpG in red and unmethylated CpG in blue). (**G**) The 79-kilobase region spanning the promoter region, the imprinted region, and the pathogenic variant on exon 17 demonstrated that the germline variant (denoted by black lines) segregated on the same haplotype as the region of intron 2 that is methylated (in red), verifying maternal inheritance.

To assess the performance of ONT targeted long-read adaptive sequencing to phase germline variants and identify allele-specific CpG methylation in the *RB1* gene to determine parent of origin, we enrolled participant 1, a 2-year-old girl with known maternally inherited RB. Participant 1 had been molecularly diagnosed in utero with an amniocentesis with the same germline variant in *RB1* [NM_000321.2:c.1625T>A; p.(Leu542Ter)] as her mother. Participant 1 had clinically evident bilateral disease at 2 weeks of age with macular and extramacular intraocular tumors requiring treatment (Figure 1, A–D). We prepared a long-read sequencing library from high–molecular weight genomic DNA (gDNA) extracted from 200 microliters of peripheral blood. Sequencing was performed on an ONT MinION R9 flow cell targeting the *RB1* gene locus and 50 kb on either side using adaptive sampling (31). Real-time base calling was carried out using the “super-accurate” model parameters with methylation calling enabled on a custom Linux-based computing workstation equipped with 2 NVIDIA RTX A6000 graphics cards and AMD Treadripper Pro 4995WX 64-core, 128-thread desktop processor. This led to complete genomic (Figure 1E) and epigenomic (Figure 1F) base coverage of the *RB1* locus. We examined the 79 kb region that encompassed the promoter region, the DMR on intron 2, and the pathogenic SNV on exon 17. This region was fully phased and the SNV segregated on the same haplotype as the methylated imprinted region on intron 2, confirming maternal inheritance of the germline variant (Figure 1G).

**Figure 2:**
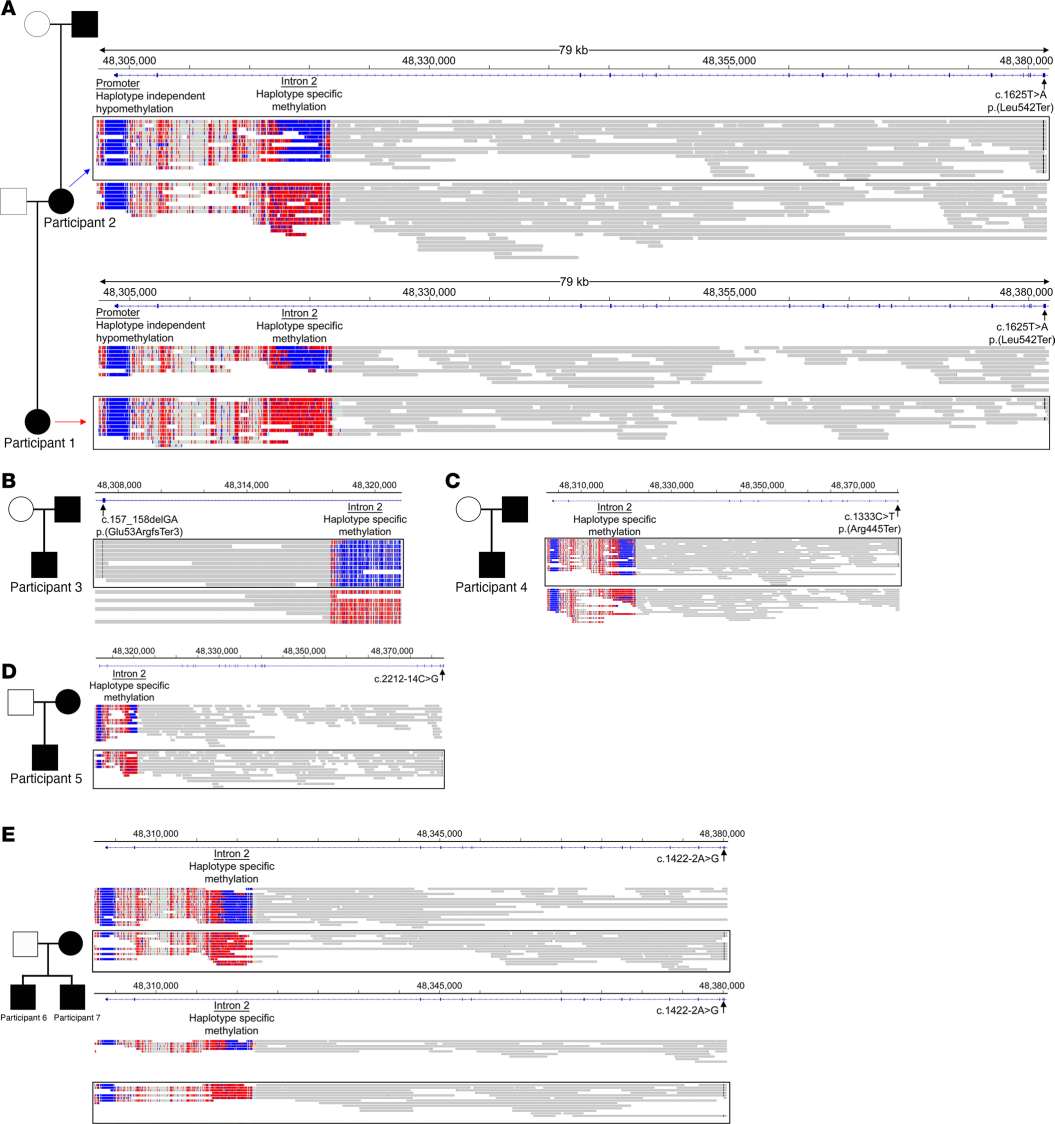
Phasing of germline variants in *RB1* and the allele-specific methylation signal correctly identifies parent of origin in known familial cases of RB. (**A**) In a family where participant 1 (maternal inheritance) and participant 2 (paternal inheritance) display different parent of origin of the same germline variant, we show that in participant 1 the germline variant (denoted by black lines) lies on the same allele harboring the methylated signal at the DMR of intron 2 of *RB1*, confirming maternal inheritance, whereas in participant 2 the germline variant (denoted by black lines) lies on the same allele harboring the unmethylated signal at the DMR on intron 2 of *RB1*, verifying paternal inheritance. In 2 cases of paternal inheritance (**B** and **C**) the germline variant (black lines) in *RB1* segregates on the same allele as the unmethylated signal at the DMR on intron 2 of *RB1*, whereas in 3 cases of maternal inheritance (**D** and **E**) the germline variant (black lines) in *RB1* segregates on the same allele as the methylated signal at the DMR on intron 2 of *RB1*.

We then benchmarked the performance of this approach in 6 participants who had previously undergone Clinical Laboratory Improvement Amendments–approved (CLIA-approved) clinical molecular testing with a known RB familial inheritance pattern. This allowed evaluation of targeted long-read sequencing on the ONT platform against best current practices for diagnostics. We sequenced participant 2, who was the mother of participant 1 and had inherited RB from her father. Our approach showed that whereas the germline variant in participant 1 segregated on the allele methylated on intron 2 (maternal inheritance), the same germline variant segregated on the allele unmethylated on intron 2, indicative of paternal inheritance, in participant 2 (Figure 2A). Further examination of 5 probands from 4 families with known familial inheritance indicated that in the 2 cases of paternal inheritance, the germline variant segregated on the unmethylated allele on intron 2 (Figure 2, B and C), whereas in the 3 cases of maternal inheritance, the germline variant segregated on the allele methylated on intron 2 (Figure 2, D–E). These studies demonstrated that in 7 cases of known familial inheritance of *RB1* variants, phased long-read genomic and epigenomic sequencing from the proband alone provided parent of origin of the affected allele without need for testing of familial DNA. We extended this approach to resolve parent of origin in 7 participants with de novo pathogenic germline variants in *RB1*, which account for the majority of heritable cases of RB.

### Phasing genomic and epigenomic signals identifies parent of origin in simple and complex de novo heritable cases of RB.

**Figure 3:**
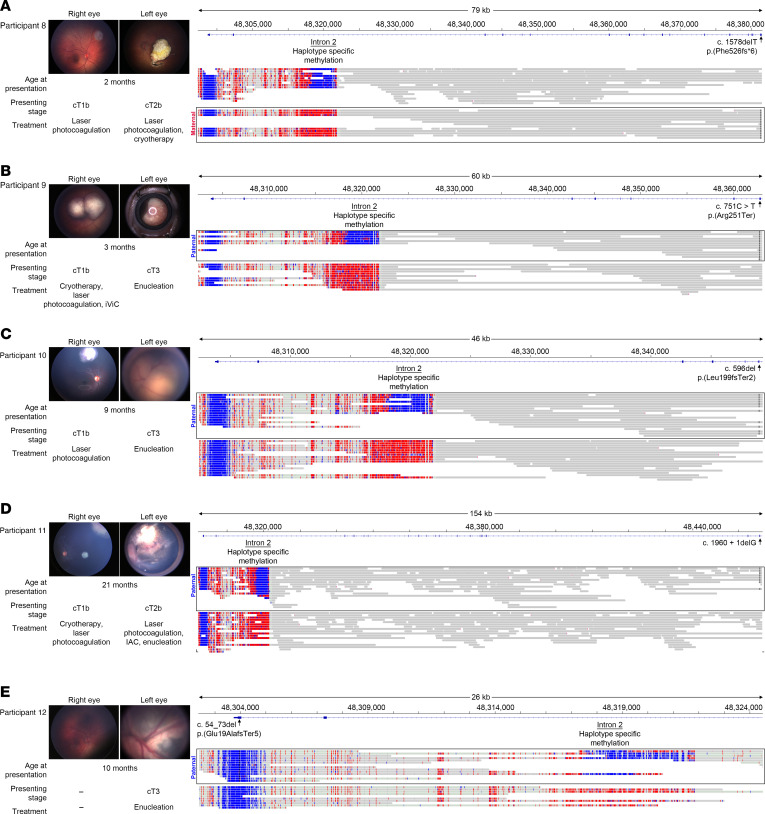
The clinical presentation and disease parameters are outlined in 5 sporadic cases of RB due to *RB1* germline variants. Targeted long-read sequencing allowed us to demonstrate parent of origin in each case, with (**A**) the germline variant (denoted by black lines) segregating on the allele with methylation (red) of the DMR on intron 2 exhibiting maternal inheritance in 1 case with good treatment response, compared with (**B**–**E**) the germline variant (denoted by black lines) segregating on the unmethylated allele (blue) of the DMR on intron 2 exhibiting paternal inheritance in the other 4 cases that all required enucleation. IViC, intravitreal chemotherapy injection; IAC, intra-arterial chemotherapy.

We examined 5 germline cases with no family history of RB that harbored a small variant, such as an SNV or insertion/deletion (indel), that had been identified from CLIA-approved clinical molecular testing. We were able to phase the germline variant with the DMR on intron 2 in each participant despite the regions being separated by up to a 154 kb distance to identify the parent of origin. In 1 case, participant 8, where the germline variant resided on the maternal allele (Figure 3A), the participant had well-controlled disease activity with local ocular therapy despite initially presenting with bilateral disease. In comparison, in the remaining 4 cases, participants 9–12, the germline variant resided on the paternal allele, and in each case, enucleation was required either at presentation or after treatment failure for disease control (Figure 3, B–E).

Complex cases such as somatic mosaicism and SVs can delay diagnosis because of added complexity of analysis required. In the case of participant 13, who presented with unilateral RB, the initial clinical genetic test result was reported as negative and then amended to include the identification of a mosaic variant 4 months later at an estimated variant allele fraction (VAF) of 12.5%–15.5%. The ability to selectively focus sequencing depth to a genomic region of interest with adaptive sequencing (31, 32) allowed us to sample the *RB1* locus at increased depth and reliably call somatic variants in a single sequencing run. We identified the *RB1* variant [NM_000321.2:c.1072C>T, p.(Arg358Ter)] at a VAF of 16% (Supplemental Figure 1A; supplemental material available online with this article; https://doi.org/10.1172/jci.insight.188216DS1), consistent with previous clinical tests, both of which were calculated from peripheral blood mononuclear cells. Furthermore, when we examined the methylation signal of the imprinted locus and the mosaic variant, 1 of the 5 reads that contained the variant encompassed the 50 kb region containing the DMR on intron 2 and the variant on exon 11 to provide evidence of maternal inheritance (Supplemental Figure 1B). In comparison, participant 14 had a complicated past medical history in addition to bilateral RB. An extensive clinical genetic testing workup was completed, including a karyotype that revealed an apparently balanced translocation between chromosome X and chromosome 13 [46,XX,t(X;13) (p22.1;q14.1)], and though it was noted that the *RB1* gene was unlikely to be disrupted at the breakpoint, it was hypothesized that its function may be disrupted (33). To capture this change, we carried out long-read sequencing targeting the entirety of chromosome 13 to capture any potential breakpoints. We were able to detect the precise breakpoint at single-base resolution on chromosome 13 utilizing local de novo assembly with DeBreak (34). Moreover, long reads were able to phase the 14-megabase region between the breakpoint location in intron 41 of the Neurobeachin (*NBEA*) gene and the DMR of *RB1* to demonstrate that the translocation was paternally inherited, and in addition, there was aberrant methylation of the *RB1* promoter on the paternal allele, likely leading to silencing as the cause of RB in this complex case (Supplemental Figure 2A).

**Figure 4:**
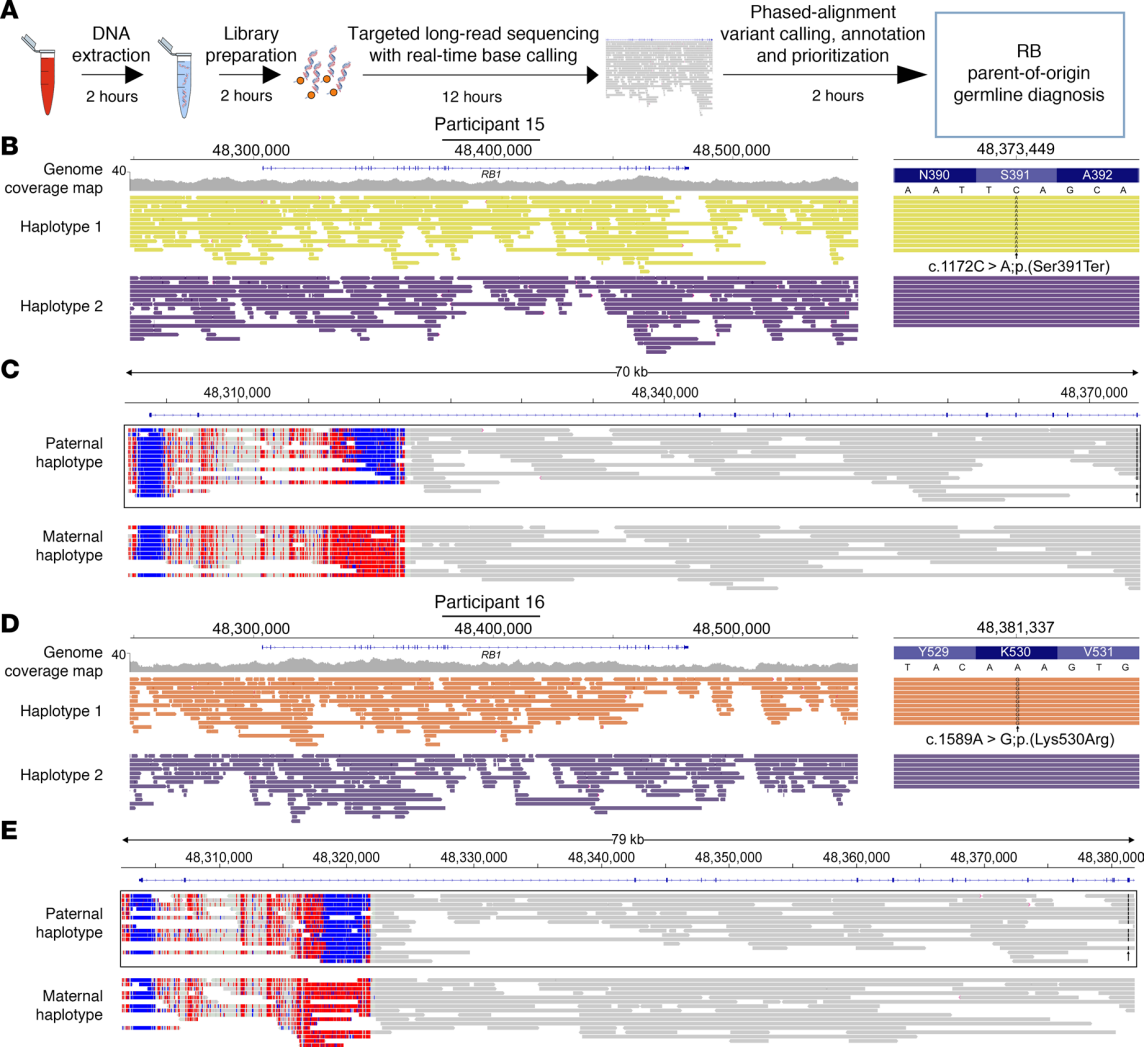
Targeted long-read sequencing can provide rapid parent-of-origin diagnosis in de novo cases of RB. (**A**) Schematic of the steps leading to germline RB diagnosis within 18 hours from receipt of blood sample in the laboratory. (**B**) In participant 15 complete phased genomic coverage allowed identification of a pathogenic variant, NM_000321.2:c.1172C>A; p.(Ser391Ter), on exon 12 of *RB1*. (**C**) Phasing of the germline variant (denoted by black lines with an arrow) with the DMR on intron 2 demonstrated that this variant resided on the paternally inherited unmethylated allele. (**D**) Similarly in participant 16 complete phased genomic coverage allowed identification of a pathogenic variant, NM_000321.2:c.1589A>G; p.(Lys530Arg), on exon 17 of *RB1*. (**E**) Phasing of the germline variant (denoted by black lines with an arrow) with the DMR on intron 2 demonstrated that this variant resided on the paternally inherited unmethylated allele.

A distinct advantage of targeted adaptive sequencing is that it provides the adequate read depth and ability to phase variants to identify pathogenic variants rapidly. We examined our sequencing run data for participants 1–12, who harbored small heterozygous pathogenic variants in *RB1*, and found that we had identified and haplotyped the disease variant within 12 hours in each case. Therefore, to test the ability of this approach to diagnose newly diagnosed cases of presumed germline RB, we carried out genomic DNA extraction, library preparation, and sequencing over 12 hours, targeting a panel (Oncoplex) (35) of 377 cancer-causing genes (to identify any potential non-*RB1* causative variants) and chromosome 13 (to capture any large SVs affecting *RB1*) for 12 hours. We analyzed the data of 2 participants who were newly diagnosed with RB and were simultaneously undergoing clinical genetic testing (Figure 4A). In both participants 15 (Figure 4, B and C) and 16 (Figure 4, D and E), we were able to identify a pathogenic variant in *RB1* and phase that with the DMR on intron 2 to show paternal origin of both variants and deliver a presumptive diagnosis within 18 hours from receipt of the blood sample.

### Parent of origin is an important prognostic biomarker for all patients with germline RB.

Determining parent of origin of germline variants in *RB1* can provide prognostic information, since variants on the paternal allele in known familial cases have been observed to have a worse clinical presentation, leading to more severe ocular disease and potential extraocular morbidity. However, since the majority of disease variants in *RB1* arise de novo, it is impossible with current clinical testing to provide prognostic insight to the majority of patients and families. To assess the clinical impact of parent-of-origin diagnosis, we analyzed the clinical outcomes of the 15 probands with heritable RB in our analysis. Participant 2 (mother of participant 1) was excluded, since phenotypic and clinical characteristics at presentation and treatment carried out were not available.

**Figure 5:**
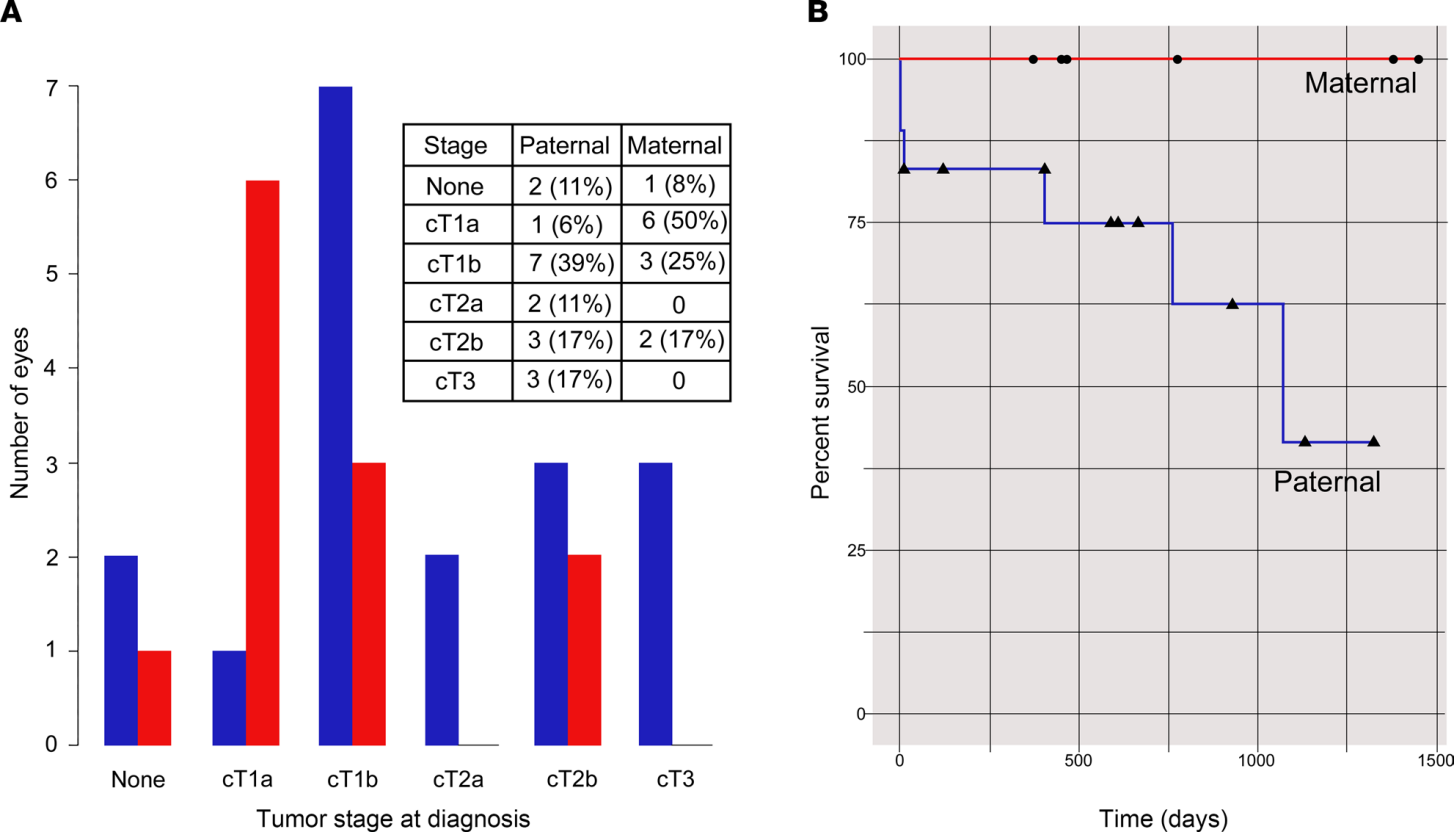
Germline variants that reside on the paternal allele in familial and de novo cases present with more advanced tumor stage and are more likely to fail chemotherapy. (**A**) Tumor stage at presentation demonstrated that more eyes with paternal inheritance (blue) had later stages of disease (CT2a, CT2b, or CT3) compared with maternal inheritance (red) eyes that had earlier stages of diagnosis (No tumor, CT1a, or CT1b). (**B**) A Kaplan-Meier survival plot of all cases of RB (familial and de novo) shows there is significantly more evidence of chemotherapy failure in eyes that exhibit paternal inheritance (*P* = 0.02).

There were 6 patients whose altered *RB1* gene was found on the maternal allele: 4 of these patients had a family history, and 2 had variants arise de novo (Supplemental Table 1). Of the 9 patients whose altered *RB1* gene was found on the unmethylated paternal allele, 2 patients had a positive family history, and 7 patients had variants arise de novo (Supplemental Table 1). The average age at time of presentation was older in paternal cases (mean = 8.5 months) compared with maternal cases (mean = 2.6 months, *P* = 0.03). Eyes from paternal cases presented with later stage disease (cT2 or cT3, following the American Joint Committee on Cancer 8th Edition Staging System, ref. 36) in 44% of cases (8 of 18 eyes, Figure 5A) while eyes from maternal cases presented with later stage disease in 17% of cases (2 of 12 eyes, Figure 5A).

Although paternal parent of origin exhibited a more advanced stage at initial diagnosis, a Fisher exact test comparing early (0/cT1) with later presenting eyes (cT2/cT3) showed no statistical difference in this small cohort. Eyes presenting at stage cT3 or later are routinely removed (enucleated) at diagnosis (37). All other eyes undergo some form of chemotherapy, including combinations of intravenous chemotherapy, intra-arterial chemotherapy, and intravitreal chemotherapy. When standard chemotherapy treatments fail, a decision is made to remove the eye or attempt radiation treatments (brachytherapy or external beam radiotherapy). To evaluate the clinical prognosis for eyes and treatment, we evaluated the survival of eyes based on whether they were enucleated or needed radiation treatment after standard chemotherapy failure. At the most recent follow-up, of the 30 eyes with heritable RB included in this study, 4 were enucleated; 2 patients elected to proceed with brachytherapy; and all 6 of these failed eyes had paternal inheritance. A Kaplan-Meier survival curve demonstrates the chemotherapy failure of eyes in this study (Figure 5B). Participants in our study with paternal allele of origin demonstrated significantly worse clinical outcomes (log-rank, *P* = 0.02).

## Discussion

Parent-of-origin effects do not follow classical Mendelian inheritance patterns, so it is necessary to identify which parent contributed the specific disease-causing allele. This requires access to familial DNA samples, but in cases of de novo variation, which are a major cause of monogenic and complex genetic diseases (38), familial DNA is not instructive to determine parent of origin. Here, we demonstrate that targeted long-read adaptive sequencing on the ONT platform allows simultaneous genomic and epigenomic profiling to determine a germline variant and phase that with an imprinted signal on the *RB1* gene that is the DMR on intron 2, to provide parent of origin in all cases of heritable RB. The focused depth of targeted genomic sequencing data obtained from adaptive sampling coupled with CpG methylome data from a single sequencing run validated prior clinical testing of germline variants and parent of origin in known cases. More importantly, this approach provided parent-of-origin diagnosis in previously unknown cases with de novo pathogenic variants in *RB1*, which account for the majority of heritable cases of RB. Additionally, in rare cases such as somatic mosaicism that required tiered clinical testing leading to prolonged diagnosis, our targeted approach was able to provide enhanced depth of coverage of the *RB1* locus to identify the variant at a VAF of 16% as well as that the mosaic variant resided on the maternal allele, all from a single sequencing run. We show that parent-of-origin assignment in RB has potential clinical implications if the germline variant resides on the paternal allele, which may lead to increased tumor burden in the eye and a higher chance to fail treatment. Together, this establishes targeted long-read sequencing as the preferred method for assignment of parent of origin and disease diagnostics.

A major advance in this work is the ability to phase a germline variant with an imprinting signal in a single sequencing run. The discovery of the imprinted region in *RB1* was first described in 2009. However, phasing of the germline variant and the differentially methylated signal of the imprinted region in intron 2 of *RB1* was not possible because of technical limitations inherent to short read lengths of clinical sequencing methodologies. In our cases of 16 participants with germline pathogenic variants in the *RB1* gene, the nearest neighboring heterozygous variant was several kilobases away. Thus, even with the benefit of clinical testing of family members, the parent of origin could not be assigned because of the long read lengths necessary to phase parent-of-origin benefits. Furthermore, since this approach is able to fully phase the differentially methylated signal with the germline variants with single or linked reads, it overcomes the ambiguity in phasing that can arise because of chromosomal recombination (39). We hypothesize that parent-of-origin assignment of germline variation in RB may not only be prognostic for disease severity in the eye but also modulate the potential risk of extraocular disease (40). Whereas this work highlights the importance of assigning germline parent of origin of pathogenic variants in *RB1* for ocular disease in the pediatric population, it will be important to apply this to better understand the role of parent of origin in adults with secondary malignancies arising from germline *RB1*. We show in an adult patient with de novo germline RB who exhibited extraocular parotid tumors in adulthood that our approach revealed that the germline variant in *RB1* in this participant arose from the paternal allele. We show in this participant that even with whole-genome-level coverage, parent of origin cannot be identified since chromosomal phase–linked methylation status is unavailable from a single short-read sequencing run (Supplemental Figure 3).

The ability to phase large regions of the genome with this approach will be critical in determining the role of parent of origin in other disease-causing genes. Only a small number of genes are known to be imprinted (41). We demonstrate in a case of a large SV in participant 14 that the DMR of *RB1* can be used to phase a distant pathogenic SV breakpoint in the *NBEA* gene (Supplemental Figure 2A). Although less than 1% of all genes in the human genome are imprinted, genes that are not differentially methylated can still display parent-of-origin effects. We show that long reads obtained from our sequencing runs allow us to phase differentially methylated genomic regions with disease-causing nonimprinted genes to assign parent of origin. We show this for the *BRCA2* gene, which like *RB1* is one of most commonly affected cancer predisposition genes in the affected pediatric population (42), and in some populations has been shown to have a parent-of-origin effect with paternal origin of mutation affecting breast cancer penetrance (43). We show that the 17-megabase region containing *BRCA2* and *RB1* can be phased to identify maternal and paternal haplotypes for parent-of-origin analysis (Supplemental Figure 2B).

This work demonstrates parent of origin in disease can be resolved by phasing a disease variant with a differentially methylated signal in the genome, either local or distant, with a targeted long-read sequencing approach. The bioinformatic tunability of adaptive sampling would allow this to be adapted to any disease loci to focus sequencing depth on the region of interest to assign parent of origin. Whereas the majority of disease diagnostics has been focused on genomic variant identification, there is emerging evidence that epigenetic markers are also critical prognostic indicators of disease severity. Recent evidence demonstrates an epigenetic signature that is predictive of survival in glioblastoma that could change treatment to ensure maximal surgical resection for better disease control (44). Similarly in RB, there is mounting evidence that methylation signals of RB tumor samples obtained through aqueous humor liquid biopsy can be predictive of disease burden and treatment response (45). Providing parent of origin at initial diagnosis would reveal another precise biomarker of risk stratification to better guide care and understand burden of disease for proper surveillance and treatment regimens. A multiomic approach can reveal the molecular basis of disease in heritable RB in one cost-effective sequencing run. Implementation of this in clinical practice can have a substantial impact by providing a more complete diagnostic outlook for children and their families with RB to better guide management and therapy in a more personalized manner.

## Methods

### Sex as a biological variable.

Both male and female participants were enrolled in this research study.

### Participant identification, recruitment, and clinical data collection.

Participants were identified based on their clinical diagnoses of RB based on history and ophthalmology findings, and all participants were undergoing an exam under anesthesia at the time of recruitment. All participants signed an informed consent document before their participation in the study. The clinical characteristics of these patients were examined, and the following variables were collected for each patient: sex, age at presentation, known familial inheritance pattern (parent-of-origin), CLIA genetic testing results of *RB1*, laterality of intraocular tumors at presentation, intraocular classification of RB at presentation, treatment, response to standard chemotherapy, and globe survival. Detailed information of all participants is listed in Supplemental Table 1.

### gDNA preparation for long-read sequencing.

A venipuncture blood of about 2 mL was obtained from study participants during routine exams under anesthesia, and gDNA was isolated using the MagAttract High Molecular Weight genomic DNA isolation kit (QIAGEN). Approximately 1 microgram of gDNA was used to make sequencing libraries using the ONT Ligation Sequencing Kit (SQK-LSK110) with slight modifications of the manufacturer’s protocol. As a modification to these instructions, 1.5 times the suggested amount of AMPure XP beads were used, and 80% (instead of 70%) ethanol was used for the bead-washing steps. During the adapter ligation and cleanup step, the Long Fragment Buffer was used to enrich DNA fragments greater than 3 kilobases in length. Approximately 50 femtomoles of DNA was loaded onto an R9.4.1 flow cell running on an ONT MinION Mk1B device.

### Long-read targeted enrichment.

A GPU-accelerated version of guppy (v6.0.7; API version 10.1.0) was used for base calling in real time on 2 NVIDIA RTX A6000 GPUs using the “super-accurate” model parameters. Target regions were enriched using Readfish (31) adaptive sampling technology implemented during real-time sequencing. To perform adaptive sampling for in silico enrichment, we prepared a BED file of each targeted locus or loci with a 50 kb buffer on each side. Sequencing experiments were run for up to 72 hours or until all the pores were inactive.

### Genomic variant and CpG methylation calling.

FASTQ files were generated using Dorado (version 7.1.4) with super-accurate model parameters and aligned to the GRCh38 assembly using minimap2. Unaligned and off-target reads were removed from the SAM file, which was converted to binary and sorted using samtools view and sort. The resulting BAM file was collated, duplicates were marked, and the reads were filtered for a minimum alignment quality score of MAPQ 50. Secondary, supplementary, and optical duplicates were removed, and then the file was indexed (collate, fixmate, sort, markdup, view, index). Small variants (SNVs and indels) were called using PEPPER with the R9 super-accurate model, and haplotyping was achieved using Margin. SVs were analyzed with DeBreak (34). The DeepVariant pipeline was used to generate a phased variant call file (VCF) (46). The VCF files were then annotated with haplotype and phase-block information, variant depth, variant quality, variant effect predictor annotations, ClinVar clinical significance, allele frequency obtained from Genome Aggregation Database, and Combined Annotation-Dependent Depletion (47) score to aid in analysis and prioritization of candidate variants. Base-level CpG methylation was called using Dorado (super-accurate 5-mC and 5-hmC model), and the percentage of methylation at CpG islands was calculated from alignment pileups at these locations.

### Statistics.

Continuous data were analyzed using a 2-tailed Student’s *t* test. Categorical data were analyzed using a 2-tailed Fisher’s exact test. Kaplan-Meier survival curves were compared using a log-rank test. An α-level of 0.05 was used for significance testing. All analyses were performed using R.

### Study approval.

Study participants were consented for genome sequencing under a protocol approved by the institutional review board (IRB) at the University of Washington, Seattle, Washington, USA (STUDY00014158). Written informed consent was obtained from all study participants or parental guardians. Experiments were conducted according to the principles expressed in the Declaration of Helsinki.

### Data availability.

The genome variant data in this study are included within the published article. Genome-sequencing data are not publicly available because of privacy and patient anonymity issues. Access to deidentified genome-sequencing data will require an IRB-approved collaboration and Data Usage Agreement. Values for all data points in graphs are reported in the Supporting Data Values file.

## Author contributions

AWS and DM designed research. AWS, KN, JH, and DM performed research. KN and DM contributed new reagents/analytic tools. AWS, KN, JH, JS, NW, EEC, RNVG, and DM recruited participants and analyzed data. AWS, RNVG, and DM wrote the paper. All authors reviewed the manuscript.

## Supplementary Material

Supplemental data

ICMJE disclosure forms

Supporting data values

## Version 1 12/26/2024

In-Press Preview

## Version 2 02/24/2025

Electronic publication
