# Supplementary material for: Prognostic importance of direct assignment of parent of origin via long-read genome and epigenome sequencing in retinoblastoma
Source: JCI Insight. 2024 Dec 26;10(4):e188216. doi: 10.1172/jci.insight.188216 (PMC11949030; doi:10.1172/jci.insight.188216)
Supplement: Supplemental data [file jciinsight-10-188216-s009.pdf]

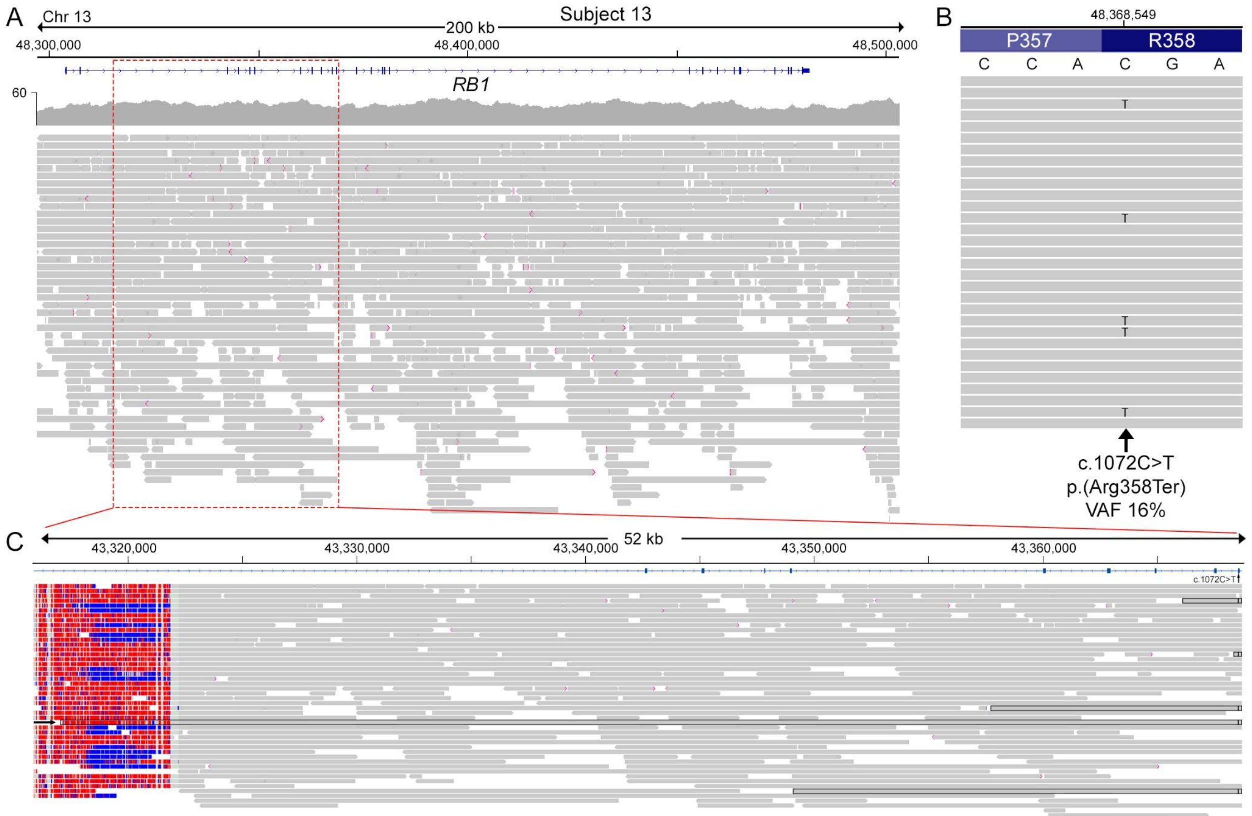

Supplemental Figure 1. Focused depth of sequencing of targeted long-read sequencing can reliably identify mosaic variants. (**A**) Our targeted approach was able to provide enhanced depth of coverage of the *RB1* locus and (**B**) identified the variant at a VAF of 16% consistent with previous clinical testing. (**C**) Furthermore, when we examined the methylation signal of the imprinted locus and the mosaic variant, one of the five variants fell on a read that encompassed the 50-kb region of the methylation signal in intron 2 and the variant to provide evidence of the allele residing on the maternal allele. Most importantly, we identified and phased the variant within days of sample receipt.

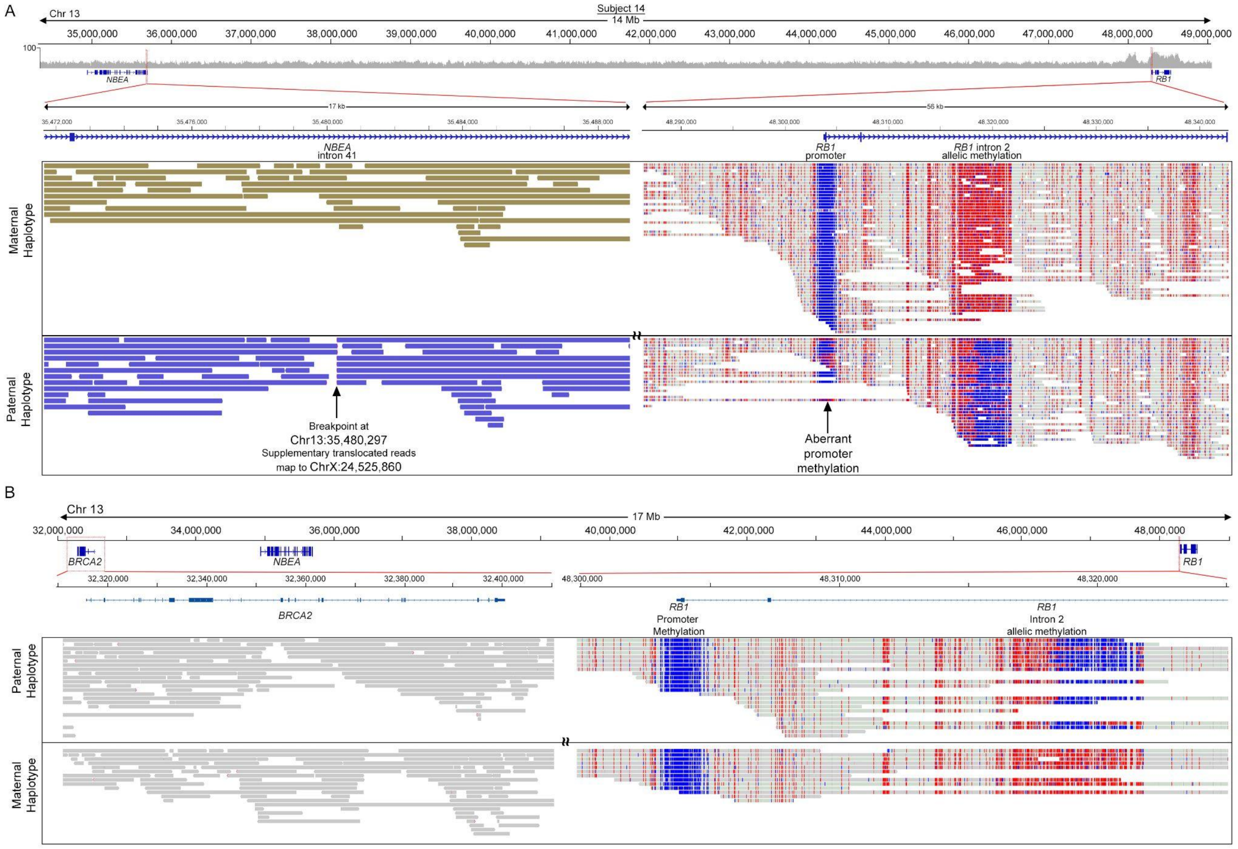

Supplemental Figure 2. Allele-specific methylation signature in *RB1* provides phasing of a distant structural variant on chromosome 13 to identify parent-of-origin in a complex case of RB. (**A**) Long-read sequencing targeting the *RB1* locus and chromosome 13 allowed detection of the precise breakpoint at single base resolution on chromosome 13 utilizing local *de novo* assembly with DeBreak. Moreover, long-reads spanned the 14 Megabase region between the breakpoint location in intron 41 of the *NBEA* gene and the DMR region of *RB1* to demonstrate that the translocation was paternally inherited. Compared to the region of intron 41 of the *NBEA* gene with reads spanning the entire region on the maternal haplotype, on the paternal haplotype we observed there was an abrupt ending of reads in intron 41 at Chr13: 35,480,297 because the supplementary reads at that point were better mapped to ChrX, indicative of a translocation between Chr13 and ChrX leading to (46,XX,t(X;13)(p22.1;q14.1)). Furthermore, we noted there was aberrant promoter methylation on the paternal haplotype. Instead of a uniformly unmethylated RB1 promoter on that haplotype, we noted hypermethylation, which would be indicative of possible X inactivation due to the translocation. (**B**) Extending the region to 17 Mb allows the ability to phase the *BRCA2* gene into maternal and paternal haplotypes by utilizing the imprinted signal in *RB1.*

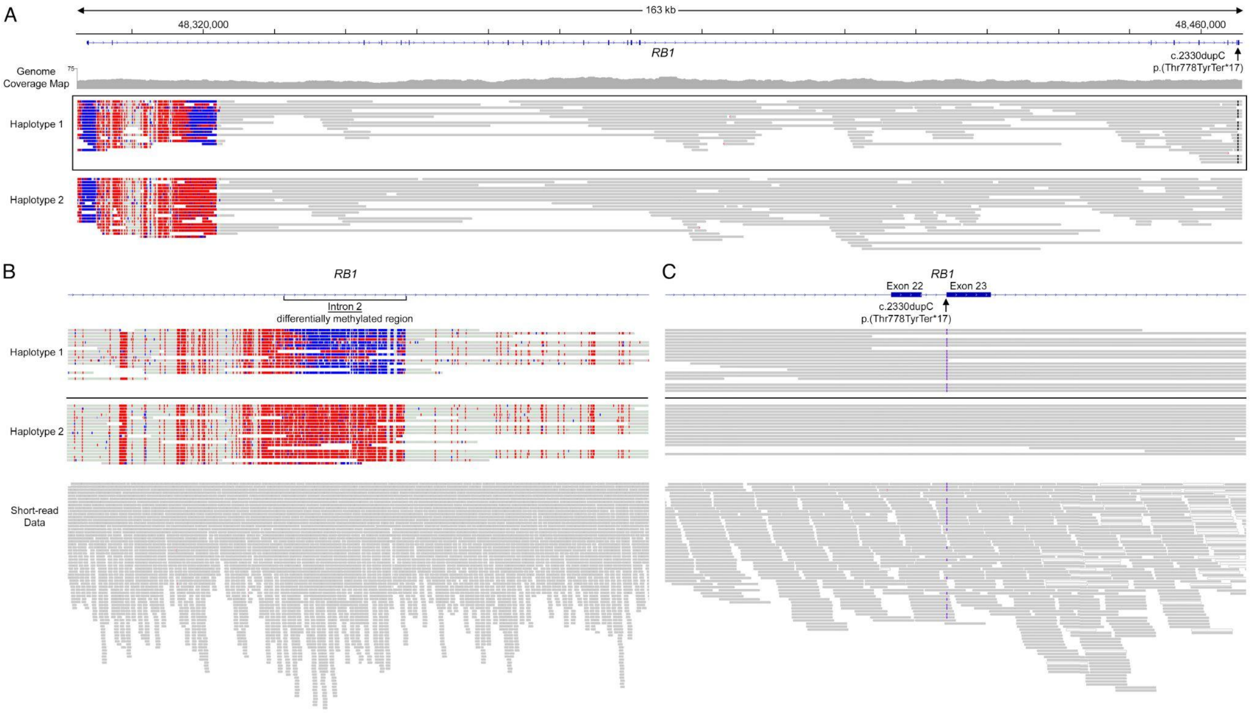

Supplemental Figure 3. Allele-specific methylation signature in *RB1* can be indicative of risk of potential secondary malignancies and are better identified by targeted long-read sequencing. (**A**) Targeted long-read sequencing of the *RB1* locus demonstrated that the disease-causing variant was on the allele that was unmethylated in the imprinted region in this subject, indicative of paternal inheritance of disease. (**B**) Examination of genome sequencing data of the DMR in intron 2 demonstrates clear signal differentiation on long-read sequencing that cannot be captured with standard short-read sequencing shown below. (**C**) In the region of the disease-causing variant, the read lengths from long-read sequencing demonstrate that the variant lies on a specific haplotype, whereas in short-read sequencing shown below of the region, the only fact that can inferred is that the variant is a heterozygous variant with no indication of its haplotype-specific nature of disease.

Subject	Sex	Age*	Familial inheritance	Long-read parent-of-origin	Germline *RB1* variant (NM_000321.2)	Disease laterality	Intraocular Classification of Retinoblastoma		Systemic treatment	Right eye treatment	Left eye treatment
1	F	0.5	Maternal	Maternal	c.1625T>A p.(Leu542Ter)	Bilateral	Right: cT1a	Left: cT1b	IVC	Laser	Laser
2	F	N/A#	Paternal	Paternal	c.1625T>A p.(Leu542Ter)	Bilateral	Unknown	Unknown	Unknown	Unknown	Enucleation
3	M	0.75	Paternal	Paternal	c.157_158delGA p.(Glu53ArgfsTer3)	Bilateral	Right: cT1a	Left: cT1b	IVC	IAC, Chemo plaque, radioactive plaque	Enucleation
4	M	0.1	Paternal	Paternal	c.1333C>T p.(Arg445Ter)	Bilateral	Right: cT1b	Left: cT1a	IVC	Laser, IAC, Cryotherapy, Radioactive plaque	Laser, IViC, Cryotherapy
5	M	3	Maternal	Maternal	c.2212-14C>G	Bilateral	Right: cT1a	Left: cT1b	-	Laser	IAC, Laser
6	M	0.75	Maternal	Maternal	c.1422-2A>G	Bilateral	Right: cT1a	Left: cT1a	-	Laser	Laser, Cryotherapy
7	M	0.37	Maternal	Maternal	c.1422-2A>G	Bilateral	Right: cT1b	Left: cT1a	IVC	Laser, IAC	Laser, IAC
8	F	2	-	Maternal	c.1578delT p.(Phe526fsTer6)	Bilateral	Right: cT1b	Left: cT2b	IVC	Laser	Laser, Chemotherapy
9	F	3	-	Paternal	c.751C>T p.(Arg251Ter)	Bilateral	Right: cT1b	Left: cT3	IVC	Laser, Cryotherapy, IViC	Enucleation
10	F	9	-	Paternal	c.596del p.(Leu199fsTer)	Bilateral	Right: cT1b	Left: cT3	IVC	Laser	Enucleation
11	M	21	-	Paternal	c.1960+1delG	Bilateral	Right: cT1b	Left: cT2b	IVC	Cryotherapy, Laser	Laser, IAC, Enucleation
12	M	10	-	Paternal	c.54_73del p.(Glu19AlafsTer5)	Unilateral	Right: None	Left: cT3	IVC	-	Enucleation
13	M	8	-	Maternal	c.1072C>T p.(Arg358Ter); 12-15% VAF	Unilateral	Right: None	Left: cT2b	-	-	IAC, Laser, IViC
14	F	5	-	Paternal	large rearrangement (chr13:chrX)	Bilateral		Left: cT1b	IVC	Laser	Laser, IAC, Cryotherapy
15	M	11	-	Paternal	c.1172C>A p.(Ser391Ter)	Bilateral	Right: cT2b	Left: cT2b	IVC	Laser	Laser
16	M	14	-	Paternal	c.1589A>G p.(Lys530Arg)		Right: cT2a	Left: cT1b	IVC	Laser	Laser

Supplemental Table 1. Clinical and genotypic characteristics of study subjects.

*age in months at first tumor presentation

#Unknown age of first tumor presentation (mother of Subject 1)

Abbreviations: intravenous chemotherapy (IVC); intra-arterial chemotherapy (IAC); intravitreal chemotherapy injection (IViC)
